# Arsenic and Other Elemental Concentrations in Mushrooms from Bangladesh: Health Risks

**DOI:** 10.3390/ijerph15050919

**Published:** 2018-05-04

**Authors:** Md Harunur Rashid, Mohammad Mahmudur Rahman, Ray Correll, Ravi Naidu

**Affiliations:** 1 Global Centre for Environmental Remediation (GCER), The University of Newcastle, University Drive, Callaghan, NSW 2308, Australia; mdharunur.rashid@uon.edu.au (M.H.R.); mahmud.rahman@newcastle.edu.au (M.M.R.); 2 Cooperative Research Centre for Contamination Assessment and Remediation of the Environment (CRC-CARE), Callaghan, NSW 2308, Australia; 3 Soil Science Division, Bangladesh Agricultural Research Institution (BARI), Joydebpur, Gazipur 1701, Bangladesh; 4 Rho Environmetrics, Highgate, SA 5063, Australia; rho.environmetrics@bigpond.com

**Keywords:** mushroom, arsenic, heavy metals, health risk, daily intake

## Abstract

Mushroom cultivation has been increasing rapidly in Bangladesh. Arsenic (As) toxicity is widespread in the world and Bangladesh faces the greatest havoc due to this calamity. Rice is the staple food in Bangladesh and among all the crops grown, it is considered to be the main cause of As poisoning to its population after drinking water. Consequently, rice straw, an important growing medium of mushrooms in Bangladesh, is known to have high As content. The objective of this study was, therefore, to determine the concentrations of As in mushrooms cultivated in Bangladesh and to assess the health risk as well. It also considered other elements, including Cd, Cr, Co, Cu, Pb, Mn, Hg, Ni, and Zn concentrations in mushrooms from Bangladesh. The mean concentrations (mg/kg) of As, Cd, Cr, Co, Cu, Pb, Mn, Hg, Ni, and Zn in mushrooms were 0.51, 0.38, 0.28, 0.01, 13.7, 0.31, 11.7, 0.12, 0.28, and 53.5, respectively. Based on the dietary intake of mushrooms, the weekly intakes of As, Cd, Cr, Co, Cu, Pb, Mn, Hg, Ni, and Zn from mushrooms for adults were 0.0042, 0.0030, 0.0024, 0.0001, 0.1125, 0.0019, 0.1116, 0.0011, 0.0023, and 0.4734 mg, respectively. Due to the low concentrations of As and other trace elements observed in mushrooms from Bangladesh, as well as relatively lower consumption of this food in people’s diet, it can be inferred that consumption of the species of mushrooms analysed will cause no toxicological risk.

## 1. Introduction

Geogenic arsenic (As) contamination in groundwater occurs in more than 107 countries worldwide [1]. About 100 million people of Bangladesh and the state West Bengal of India together [2], and more than 296 million of the global population in total [1], are at risk of As poisoning. In As-prone areas, many people suffer from skin disorders, respiratory, nervous, obstetric system disorders, diabetes, cardiovascular diseases, as well as cancers of various organs, such as skin, kidney, liver, bladder, etc. [2]. Bangladesh is a densely-populated country and to cope with the demand of the burgeoning population, it has intensified rice production especially during the dry season, which provides higher a yield than rice varieties cultivated in the wet season. The dry season rice requires large amounts of irrigation water that reach as high as 11,500 m^3^/ha [3], mostly extracted from groundwater through shallow tube-wells. Unfortunately, elevated levels of naturally-originated As have been detected in groundwater of Bangladesh [4] and the As-contaminated groundwater is extensively used for irrigating crops, especially paddy rice. A study estimated that irrigation with As-contaminated groundwater introduced 1360 tons of As into paddy soil each year in Bangladesh [5], although the mass balance and fate of the introduced As in agricultural soils is yet to be determined. Rice grown in As-contaminated soil and irrigation water increase As concentration in rice grain, shoos, and straw, which are primarily used as human food and cattle feed [6]. The concentration of As exceeded the World Health Organization (WHO) guideline value (10 µg/L) for drinking water in 42% of the 52,202 tube wells in the contaminated regions [4]. Concentrations of other elements, such as Mn, Cr, Pb, and Ni also exceeded the WHO health-based drinking water guideline values in the groundwater of Bangladesh [7].

Mushrooms are heterotrophic eukaryotic organisms classified in the kingdom of fungi. There are about 5.1 million fungal species in the world [8]. Over 75,000 species of fungi exist in the European continent, of which over 15,000 species are macro fungi (fungi that form fruiting bodies or sporocarps) which are visible to the naked eye [9]. The fruiting body bears spores is the morphological part of the fungus that is commonly called mushroom. More than 2000 mushroom species prevail in nature, of which only about 22 species are cultivable [10]. They have been an important part of diet in many countries [11], especially the cultivated ones, such as *Agaricus* spp., *Pleurotus* spp., *Lentinus edodes*, *Volvariella volvacea*, and *Auricularia* spp. [12]. Across the world mushroom cultivation is now a multi-billion dollar business [13]. Commercial mushroom cultivation was initiated by the Bangladesh Agricultural Research Council in Mushroom Culture Centre at Savar, Dhaka in early 1980s [14] as Bangladesh is one of the most suitable countries for mushroom cultivation due to its tropical monsoon type climate and low production cost. Apart from Savar, mushroom cultivation has been booming near the capital and other districts of Bangladesh. In the last 20 years, mushroom consumption and production have increased at a faster rate than almost any other agricultural food products. Rice straw and sawdust are usually used as media to cultivate mushrooms in Bangladesh; of course, wheat straw, gypsum, oak and beech sawdust or chicken manure are also used as typical substrates for mushroom cultivation in many parts of the world [15,16,17].

The literature demonstrates that any of the substrates, if collected from polluted areas, can facilitate higher accumulation of trace elements, particularly As and Hg [18,19]. One study revealed that As concentration in rice straw increased significantly (from 3.9 mg/kg to 91.8 mg/kg) with increasing As concentration in irrigation water (up to 8.0 mg/L of As) [6]. In As-contaminated areas, rice straw has been found to contain 1.15 mg/kg of As [20]. Various investigations have dealt with metal concentrations of mushrooms, especially edible ones, and many edible mushroom species are known to accumulate high levels of heavy metals [21]. However, there have not been any previous studies focused on the concentrations of As and other toxic elements in edible mushrooms grown in Bangladesh. This study reports concentrations of As and other elements in edible mushrooms available near Dhaka City, Bangladesh. We use those data to assess whether mushroom consumption poses any health risks to humans, given that As, Cr, Cd, Pb, and Hg are highly toxic and the exposure route is often through food.

## 2. Materials and Methods

### 2.1. Mushroom Cultivation and Consumption in Bangladesh

There is no information available on daily consumption of mushroom/person in Bangladesh. In Bangladesh, mushroom production and consumption is limited to urban and semi-urban areas. Mushrooms are consumed by 77% of higher-income groups and by 37% of medium-income groups [22]. Before the 1970s, mushrooms were usually consumed by some of the tribes as per their custom, but now the situation has changed due to mushrooms’ agreeable aroma, excellent subtle flavour and, above all, their nutritional and medicinal values. Many exotic food preparations, such as soup, vegetables, pickles, etc., are made from mushrooms, and have become highly popular for about 58% of customers in Bangladesh [22]. According to the most recent database, about 620–675 tons of fresh mushrooms is produced in Bangladesh per annum [23], thus, the mushroom consumption rate is estimated as 0.09 g (fresh weight) weekly, considering 675 tons of mushroom production per annum and the total population for that time was 140.6 million [23]. Data from the wholefood catalog [24] indicates that the dry matter content of the mushrooms averages 13%, giving an average weekly intake on a dry matter basis of 0.01 g. Generally mushrooms from three genera, namely *Pleurotus*, *Agaricus*, and *Calocybe* are cultivated in Bangladesh at the rate of 97%, 1%, and 1%, respectively [23] and they are commonly called Oyster, Button, and Milki mushrooms, respectively. The additional 1% of mushrooms are mainly *Volvariella* spp. and *Auricularia* spp. It was also reported that among the *P.* spp., 61% were *P. ostreatus*, followed by 19% *P. sajorcaju*, 12% *P. florida*, and 8% of *P. high king*-*51* [23]. At the time of collecting fresh edible mushrooms for the present study from the Mushroom Development and Extension Institute, Savar (on the outskirts of the capital, Dhaka) and a commercial farm of Gazipur (about 10 km from the main city), only *P. ostreatus* and *P. high-king* were in the production phase.

### 2.2. Sampling, Sample Processing, Digestion and Analysis

Fresh edible mushrooms (*P. high-king* and *P. ostreatus*; *n* = 2 × 20) with respective growing media (rice straw; *n* = 2 × 20) were collected from the “Mushroom development and Extension Institute” Savar, Dhaka, and the same from a private mushroom farm at Gazipur during 2014. Some commercially-available powdered mushroom samples (*Pleurotus* sp.; *n* = 5) were also purchased from local markets in Dhaka and Gazipur.

The substrate debris and stalks of the mushroom samples were removed with a disposable plastic knife. The mushroom samples were then washed in running tap water (three times) to remove the debris followed by washing three times with deionised water. The washed samples were then air dried for approximately 48 h at 20 °C and then placed in an oven for drying at 60 °C. The dried samples were then homogenized and ground. A similar procedure was also employed for the processing of growing media.

### 2.3. Sample Preparation

A microwave digestion system with 40 rotors (model: MARS 6, CEM Corporation, Matthews, NC, USA) was used for the digestion of the samples. Trace grade concentrated nitric acid (Fisher Chemicals, Hampton, NH, USA) was used for the digestion of mushroom. The As speciation study was conducted after extracting samples with 2 M trifluoroacetic acid (TFA) as per standard procedure [6].

### 2.4. Sample Analysis

An Agilent 7500ce (Agilent Technologies, Tokyo, Japan) inductively-coupled plasma mass spectrometer (ICP-MS) was used to determine the amount of As and other elements in mushrooms and rice straw. An Agilent liquid chromatography system (1100 series, Agilent Technologies, Tokyo, Japan) was used for As speciation study. A Hamilton PRPX-100 along with a guard column coupled with ICP-MS was used to measure As species (As(III), As(V)), MMA(V), and DMA(V) in mushroom. The elemental concentration of samples was determined on a dry weight basis.

### 2.5. Quality Control: Analysis of Standard Reference Material (SRM)

Standard reference material (SRM) from the National Institute of Standards and Technology (NIST), such as 1573a (tomato leaves), were analysed for As and other elements utilizing the same procedure as that used for mushroom and rice straw samples to check the effectiveness of the digestion and analytical procedure.

### 2.6. Health Risk Index (HRI)

The health risk index was measured as the ratio of estimated exposure of mushroom and oral reference dose [25]. Estimated exposure was obtained by dividing weekly intake of elements by their safe limits or oral reference dose. An index of more than 1.0 was considered unsafe for human health [26].

The weekly intake of elements (WI_E_) was calculated by using the following equation:

WI_E_ = C_E_ × W_MI_/B_AW_,

where C_E_, W_MI_, and B_AW_ represent the elemental concentrations in mushrooms (mg/kg), weekly intake of mushrooms, and average body weight (45 kg) of adults in Bangladesh [27].

### 2.7. Statistical Analysis

All the statistical analyses were carried out using R software [28]. The data on arsenic and heavy metals concentration in mushrooms were analysed using analysis of variance (ANOVA) using the ‘aov’ command. The residual sum of squares was then used for calculating Fisher’s protected least significant differences. The accumulation factors were assessed as the ratio of the concentration in the mushroom divided by the concentration in the medium. Correlations between concentration in the medium and concentration in the mushroom were performed using the ‘cor’ function in R. Principal component analyses were performed using the ‘princomp’ command in R and were based on the correlation matrix.

## 3. Results and Discussion

**Figure 1:**
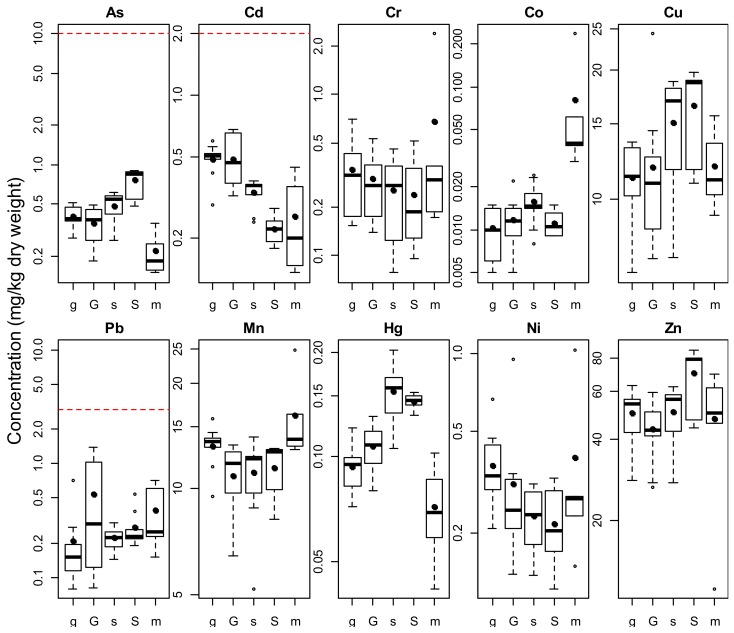
Boxplot for arsenic and metal concentrations of mushrooms with UK guideline values (red dashed lines). Legend: S = Savar (HK, *n* = 20), G = Gazipur (HK, *n* = 20), s = Savar (PO2, *n* = 20), g = Gazipur (PO2, *n* = 20), m = market basket (*n* = 5).

Table 1 shows the concentration of As and other trace elements in mushrooms under investigation. It also demonstrates the percent recovery of analytical results where we noticed about 88–112% recovery using tomato leaves (NIST SRM). Figure 1 demonstrates that As levels were higher (*p* < 0.001) at Savar (0.62 mg/kg) than at Gazipur (0.38 mg/kg) and at Savar As in *P. high-king* (0.76 mg/kg) was higher (*p* < 0.05) than the level in *P. ostreatus* (0.49 mg/kg). Cd showed the opposite trend with Cd being higher (*p* < 0.001) at Gazipur (0.49 mg/kg) than at Savar (0.28 mg/kg) where *P. ostreatus* (0.34 mg/kg) was higher (*p* < 0.05) than *P. high-king* (0.22 mg/kg). Mercury was higher (*p* < 0.001) at Savar (0.15 mg/kg) than at Gazipur (0.11 mg/kg). Concentration of Pb in *P. high-king* (0.40 mg/kg) was almost double than that of *P. ostreatus* (0.22 mg/kg) and the difference was significant (*p* < 0.05). The other elemental differences between the species were not statistically significant. The order of the median concentrations (mg/kg) of elements in mushrooms was found to be as Zn > Cu > Mn > As > Cd > Pb > Cr > Ni > Hg > Co with value of 50.4, 12.5, 12.2, 0.49, 0.30, 0.23, 0.22, 0.22, 0.12, and 0.01, respectively, for *P. high-king*. On the other hand in case of *P. ostreatus* the concentrations (mg/kg) were 54.8, 12.7, 12.6, 0.45, 0.38, 0.31, 0.29, 0.19, 0.12, and 0.01 for those elements that follows the order of Zn > Mn > Cu > As > Cd > Cr > Ni > Pb > Hg > Co, respectively.

The elemental concentrations of cultivated mushrooms in this study were compared to research findings on cultivated mushrooms by various researchers around the world (Table 2) though available data on cultivated mushrooms are less [29,30] as compared to wild ones. Reported results showed that elemental concentrations in the mushrooms varied widely depending on locations and species.

### 3.1. Arsenic

Mean concentration of total As (mg/kg, dw) in *Pleurotus high-king* and *Pleurotus ostreatus* were 0.56 ± 0.25 and 0.45 ± 0.11, respectively (Table 1 and Table 2). Noteworthy, however, is *Pleurotus high-king*, with As concentration reaching near 1.0 (0.91 mg/kg, dw) which could be considered low as those were below the normal level (<1 mg/kg, dw) stated for mushrooms grown in uncontaminated media [44,45]. Amongst different cultivated mushrooms in different countries, total As concentration of this study were lower with an exception of *Lentinula edodes* in Spain (Table 2). However, an investigation in Bangladesh found As in mushrooms below the detection level [31]. For dry mushrooms, the UK guideline value for As is 10.0 mg/kg [46] and the As concentration in the mushroom samples both from cultivated and market basket were within the safe limit according to the guideline value (Figure 1). Furthermore, the concentration of As in mushrooms of this study were within the limit of Australia and New Zealand Food Standard Code [47], and also below the maximum allowable concentrations (1.0 mg/kg) of As recommended by the Ministry of Health of China [48].

Inorganic As (arsenate and arsenite) is classified as human carcinogen [49]. Speciation of As (arsenite, arsenate, MMA, and DMA) in selected mushroom samples (*n* = 9) was conducted and found the percentage of inorganic As species ranged from 38 to 72%. This was consistent with study in which the researchers also reported that inorganic species were the major components in mushrooms [50] and ranged from 0.14 to 0.89 mg/kg of As, similar to this study. In a study in Bangladesh during 2002 scientists [6] found inorganic As species as the predominant species in rice straw which estimated arsenate, arsenite, and DMA as about 90%, up to 8% and up to 4% of the total, respectively. The inorganic species are the most harmful and potentially pose a health risk to the population if those are consumed on a regular basis through foods. Additionally, DMA is a probable human carcinogen [51,52] according to the United States Environmental Protection Agency (USEPA), though less toxic than inorganic As.

### 3.2. Cadmium

The Cd concentrations of mushrooms in this study were very low (below 1.0 mg/kg, dw). However, undetectable level of Cd was found in mushroom in a study in Bangladesh [31]. Our results were in agreement with the study conducted in Mexico [39], Brazil [36], and Australia [40], with the only exception in Mexico [39] for *P. ostreatus* mushroom (Table 2). For dry mushrooms, the UK guideline [46] value for Cd is 2.0 (mg/kg, dw) and the Cd concentrations of the mushroom samples in the current study (0.18–0.68 mg/kg) were far below the UK guideline value (Figure 1).

### 3.3. Chromium

The Cr concentrations of mushrooms in this study were much less than those in a previously-published database (Table 2) on cultivated mushrooms except a study in Bangladesh [31]. The highest concentration of chromium (63.0 mg/kg, dw) was observed in Mexico [39] in *P. ostreatus* mushroom and the lowest (4.06 mg/kg, dw) was in *A. bisporus* in the same country (Table 2). The same cultivated mushroom (*P. ostreatus*) showed significantly higher content (63.0 mg/kg, dw) in Mexico [39] than in Bangladesh.

### 3.4. Cobalt

The concentrations of Co in this study were similar (0.011 ± 0.004, 0.013 ± 0.005 mg/kg, dw) in both *P. high-king* and *P. ostreatus* which were about ten times lower in *A. bisporus* in Australia [40] and one thousand times lower than *P. sajor-kaju* in Turkey [41] (Table 2).

### 3.5. Copper

The concentrations of Cu in this study (6.7–24.3 mg/kg, dw) were slightly higher than *P. sajor-kaju*, a cultivated mushroom of Turkey [41] but highest concentration was observed in Mexico [39]. However, *P. ostreatus* of another study in Bangladesh obtained higher Cu concentration [31] than the same one of this study. *A. bisporus* in Australia [40] and Hungary [42] contained about four times higher Cu than this investigation (Table 2).

### 3.6. Lead

Lead concentrations of mushrooms found in this study were 0.40 ± 0.39 and 0.22 ± 0.13 mg/kg in *P. high-king* and *P. ostreatus,* respectively, which were slightly higher than another study conducted in Bangladesh [31] with four species including *P. ostreatus*. Cultivated *P. sajor-caju* of Turkey [41] showed about fifty time higher Pb content than our results whereas *A. bisporus* of Australia [40] demonstrated about ten and twenty times higher than *P. high-king* and *P. ostreatus* in our study, respectively. However, a study carried out in Mexico [39] found lower levels of Pb in *P. ostreatus* and *A. bisporus*. For dry mushrooms, the UK guideline value for Pb is 3.0 mg/kg [46,53]. The Pb concentration in the mushroom samples was in compliance with the UK guideline value (Figure 1). The Pb concentrations in this study were much below the maximum allowable concentrations (2 mg/kg) of Pb recommended by the Ministry of Health of China [48].

### 3.7. Manganese

A little variation was observed in Mn concentration in cultivated mushrooms of this study, e.g., 11.2 ± 2.17 and 12.2 ± 2.4 mg/kg in *P. high-king* and *P. ostreatus*, respectively (dry weight basis). The results of this study were much lower than the results in *P. sajor-caju* in Turkey [41], *P. ostreatus* in Mexico, *P*. *ostreatus, A. bisporus, V. volvacea, G. lucidum* in Bangladesh [31], and slightly higher than *A. bisporus* in Mexico [39] and Australia [40] (Table 2).

### 3.8. Mercury

Mercury is highly toxic element of the environment poses health risk depending on its form, concentration, duration, and method of exposure [54]. The concentration of Hg in the substrate is of much significance for mushroom production as a high concentration in the substrate accelerates its accumulation into the cap and stipe [18]. The Hg concentrations in mushrooms in this study were 0.126 and 0.124 mg/kg (dw) in *P. high-king* and *P. ostreatus*, respectively (Table 2,) which were much lower than that of Turkey [41] and Mexico [39] (Table 2).

### 3.9. Nickel

The Ni concentrations of mushrooms in this study were much less (0.12–0.95 mg/kg) than those reported in the literature (Table 2). High levels of Ni (31.5 mg/kg, dw) were reported in mushrooms in Turkey [41] whereas the same species, *P. ostreatus*, in this study demonstrated a maximum Ni concentration of 0.665 mg/kg.

### 3.10. Zinc

In this study the highest mean Zn level (56.9 mg/kg) was observed in *P. high-king* and the lowest (50.2 mg/kg, dw) was in *P. ostreatus*. The species *P. ostreatus* showed a higher Zn concentration (59.2 mg/kg, dw) in Ethiopia [43] and in another study in Bangladesh [31] as compared to our results. Same scenarios were also observed for *P. sajor-caju* of Ethiopia [43] and *A. bisporus* in Hungary [42] where very high differences in Zn concentration were obtained for the same species in different locations (Table 2).

### 3.11. Concentrations of Elements in Powdered Mushrooms for Sale

Ground mushroom samples (*P. ostreatus and P. high-king*) available in the market were also collected for analysis to investigate whether there is any toxicological hazard prevails for their consumption. The concentrations (range) of As, Cd, Cr, Co, Cu, Pb, Mn, Hg, Ni, and Zn in mushroom samples (*n* = 5) were 0.15–0.36, 0.14–0.44, 0.17–2.4, 0.03–0.24, 9.20–15.6, 0.15–0.61, 12.9–24.9, 0.041–0.10, 0.15–1.04, and 11.1–69.6 mg/kg, respectively, which proved that mushroom powder of the market contained lower concentrations of As, Cd, Cu, Pb, Hg, Zn, and higher concentrations of Cr, Co, Mn, and Ni compared to mushrooms directly collected from the growers in this study.

### 3.12. Elemental Accumulation

The average As, Cd, Cr, Co, Cu, Pb, Mn, Hg, Ni, and Zn concentration in the substrates (rice straw) of Savar were 0.563, 2.15, 2.05, 0.789, 6.48, 2.91, 1165, 0.134, 6.09, and 62.1 mg/kg, respectively, while the concentration of those elements in Gazipur were 0.47, 0.18, 1.27, 0.58, 8.64, 2.58, 1079, 0.148, 6.57, and 58.1 mg/kg, respectively.

The accumulation efficiency of elements in mushrooms from growing media was calculated using the accumulation factor, which is defined as the ratio of elemental concentration in the mushroom to that in the growth medium. Organisms are termed as metal hyper-accumulators when the accumulation factor remains above 1.0 [55] or, in another way, the species is termed as a hyper-accumulator when it contains more than 100 times higher concentrations of an element than other species growing on substrates of similar features [33]. The data in Table 3 demonstrated that element accumulation factors were more than 1 for Cu and Cd at both sites, and greater than 1.0 for As and Zn at Savar, but less than 1.0 in other cases (representing exclusion). It has been observed that mushroom species are able to accumulate high amounts of some elements, e.g., As, Cd, Pb, Hg, and Se [56], particularly when mushrooms are collected from sites adjacent to heavy metal smelters [57], and landfills of sewage sludge emission areas [58]. Concentrations of heavy metals were also observed high in proximity to highways with high traffic [59,60,61] and emission areas [62]. The sampling site at Savar was close to highways, industries, and brick kilns, which might pose high emission and pollution [63], whereas the Gazipur site was far away from highway traffic and industries and surrounded by large trees and vegetation. Contamination of mushrooms grown at Gazipur might, therefore, be attributed to the substrate rather than the environment.

### 3.13. Correlations between Elements in Mushrooms

There were significant statistical correlations between elemental concentrations in mushrooms which are shown in Table 4. Significantly positive correlations were established between As and Hg (*p* ≤ 0.01), Hg and Zn (*p* ≤ 0.01), Cu and Hg (*p* ≤ 0.01), Pb and Cr (*p* ≤ 0.01), Cu and Mn (*p* ≤ 0.01), Zn and Mn (*p* ≤ 0.01), Zn and Cu (*p* ≤ 0.01), As and Cu (*p* ≤ 0.01), As and Zn (*p* ≤ 0.01), Co and Cr (*p* ≤ 0.01), As and Mn (*p* ≤ 0.01), Pb and Co (*p* ≤ 0.01), and Cd and Ni (*p* ≤ 0.01). The other correlations between elements were not significant. Significant negative correlations were found between Cd and As (*p* ≤ 0.01), and Hg and Cd (*p* ≤ 0.05).

### 3.14. Correlations between Elemental Concentrations in the Medium and Mushroom

It was noted that As concentrations in the substrate for *P. high-king* in Savar were 0.660 mg/kg which was significantly higher (*p* < 0.05) than that of Gazipur (0.473 mg/kg). There was effectively no correlation between the concentrations of element in the substrate with the corresponding element in the mushroom. This finding agrees with some other studies [33,64,65].

### 3.15. Market Basket Study

A comparison of analyte levels in the market basket survey with the commercial products can be obtained from Figure 1. The As level in the market basket (0.22 mg/kg) was lower (*p* < 0.01) in the levels from the mushroom farm (0.50 mg/kg). The Hg levels were also lower (*p* < 0.001) in the market basket than in those obtained from the mushroom farms. The Co levels were higher in the market basket (0.081 mg/kg) than in the values from the mushroom farm (0.012 mg/kg).

### 3.16. Daily Consumption of As and Other Elements from Mushrooms: Health Risk Index

Based on using weekly intake rates of mushrooms and the median concentrations (fresh weight) of elements, we have estimated the weekly total consumption of As and other elements from mushrooms for adults (Table 5).

The Joint FAO/WHO Expert Committee on Food Additives [66] recommends that provisional tolerable weekly intakes (PTWI) of As, Cd, Pb, and Hg are 15, 7, 25, and 5 µg/kg/week, respectively. Therefore, the weekly tolerable intakes of As, Cd, Pb, and Hg for a person weighing 45 kg would be 0.675, 0.315, 1.125, and 0.225 mg, respectively [67]. We note that the PTWI value for As was withdrawn in 2011.

The health risk index (HRI) was estimated based on standard methods [68]. Considering the average body weight (45 kg) of an adult Bangladeshi [69], fresh mushroom consumption (0.09 g weekly) and median concentration of As, Cd, Cr, Cu, Pb, Mn, Ni, and Zn in mushroom found in this study, then the estimated HRI for As, Cd, Cr, Cu, Pb, Mn, Ni, and Zn are 0.0448, 0.0097, 0.0026, 0.0089, 0.0015, 0.0025, 0.0004, and 0.0050, respectively (Table 5). All the values of HRIs based on median concentration were less than one (closer to zero), suggesting that consumption of the investigated mushrooms do not pose any health risks from As and other elements for Bangladeshi consumers.

## 4. Conclusions

The concentrations of elements varied between mushrooms and collection sites. Mushrooms alone contributed to the intake of As and other elements which were below the PTWI values due to the very low consumption of mushrooms in the diet of Bangladeshis. Moreover, the estimated daily intakes were based on the best available production and consumption data, which was meagre. Due to the low consumption rate of mushrooms, it is, therefore, important to examine other food sources, such as rice, vegetables, etc., to assess the total exposure load from main Bangladeshi foods for the pollutants. As the tolerable/permissible values of As, Cd, and Pb are available from UK guidelines, future work should focus on formulating the values for other metals, which will provide more clear information for risk assessment.

## Figures and Tables

**Table 1:** Concentrations of elements in *Pleurotus high-king* (*n* = 40) and *Pleurotus ostreatus* (*n* = 40) mushroom samples (mg/kg, dw).

Element	Mushroom Species	Mean ± SD	Median	Range	NIST SRM 1573a (Tomato Leaves)		
					Certified Values	Observed Values	Recovery (%)
As	*P. high-king*	0.56 ± 0.25	0.489	0.18–0.91	0.112 ± 0.004	0.122 ± 0.003	109
	*P. ostreatus*	0.45 ± 0.11	0.45	0.263–0.62			
Cd	*P. high-king*	0.35 ± 0.17	0.301	0.18–0.68	1.52 ± 0.04	1.46 ± 0.060	96.1
	*P. ostreatus*	0.41 ± 0.10	0.38	0.24–0.599			
Cr	*P. high-king*	0.27 ± 0.14	0.223	0.096–0.533	1.99 ± 0.06	1.76 ± 0.050	88.4
	*P. ostreatus*	0.3 ± 0.16	0.313	0.078–0.697			
Co	*P. high-king*	0.011 ± 0.004	0.011	0.005–0.022	0.57 ± 0.02	0.61 ± 0.040	107
	*P. ostreatus*	0.013 ± 0.005	0.014	0.005–0.024			
Cu	*P. high-king*	14.2 ± 4.9	12.5	7.30–24.3	4.7 ± 0.14	5.3 ± 0.19	113
	*P. ostreatus*	13.2 ± 3.7	12.6	6.70–18.8			
Pb	*P. high-king*	0.40 ± 0.39	0.23	0.08–1.37			
	*P. ostreatus*	0.22 ± 0.13	0.194	0.079–0.72			
Mn	*P. high-king*	11.2 ± 2.17	12.2	6.45–13.4	246 ± 8	232 ± 12	94.3
	*P. ostreatus*	12.2 ± 2.4	12.7	5.1–15.8			
Hg	*P. high-king*	0.126 ± 0.03	0.12	0.11–0.144	0.034 ± 0.004	0.032 ± 0.006	94.1
	*P. ostreatus*	0.124 ± 0.03	0.121	0.094–0.154			
Ni	*P. high-king*	0.263 ± 0.18	0.22	0.12–0.95	1.59 ± 0.07	1.50 ± 0.08	94.3
	*P. ostreatus*	0.3 ± 0.12	0.292	0.136–0.665			
Zn	*P. high-king*	56.9 ± 19.5	50.4	26.3–85.8	30.9 ± 0.7	27.6 ± 0.6	89.3
	*P. ostreatus*	50.2 ± 11.3	54.8	27.5–63.2			

*P. high-king: Pleurotus high-king, P. ostreatus: Pleurotus ostreatus*.

**Table 2:** Total As concentration (mg/kg, dw) in cultivated mushrooms in this study and in literature (adapted and modified [30]).

Element	Study Location	Species	Concentration		Reference
			Mean	Range	
As	Bangladesh	*P. high-king*	0.56 ± 0.25	0.18–0.91	This study
		*P. ostreatus*	0.45 ± 0.11	0.263–0.62	
	Bangladesh	*P. ostreatus*	BDL		[31]
		*A. bisporus*			
		*V. volvacea*			
		*G. lucidum*			
	Spain	*L. edodes*	1.393		[32,33]
		*A. bisporus*	0.185		
		*P. ostreatus*	0.335		
	Canada	*A. bisporus*	0.14 ± 0.04		[34]
	Poland	*A. arvensis*		BDL	[35]
		*A. bisporus*		0.08–0.71	
		*A. Bisporus*		0.15–1.4	
	Brazil	*P. ostreatus*	0.056 ± 0.004		[36]
		*P. florida*	0.073 ± 0.018		
		*P. eryngui*	0.009 ± 0.003		
		*P. salmoneostramineus*	0.043 ± 0.004		
		*A.* sp.	0.125 ± 0.014		
		*A. bisporus*	0.097 ± 0.024		
		*L. edodes*	0.210 ± 0.009		
	Ghana	*P. ostreatus*	0.04		[37]
		*T. clypeatus*	0.1		
	India	*A. bisporus*	0.64 ± 0.16		[38]
Cd	Bangladesh	*P. high-king*	0.35 ± 0.17	0.18–0.68	This study
		*P. ostreatus*	0.41 ± 0.10	0.24–0.60	
	Bangladesh	*P. ostreatus*	BDL		[31]
		*A. bisporus*			
		*V. volvacea*			
		*G. lucidum*			
	Mexico	*P. ostreatus*	5.39		[39]
		*A. bisporus (caps)*	0.54		
	Brazil	*P. ostreatus*	0.074 ± 0.002		[36]
		*P. florida*	0.220 ± 0.013		
		*P. eryngui*	0.011 ± 0.003		
		*P. salmoneostramineus*	0.229 ± 0.004		
		*A.* sp.	<LD		
		*A. bisporus*	<LD		
		*L. edodes*	0.190 ± 0.011		
	Australia	*A. bisporus*	0.18 ± 0.02		[40]
Cr	Bangladesh	*P. high-king*	0.27 ± 0.14	0.096–0.53	This study
		*P. ostreatus*	0.30 ± 0.16	0.078–0.70	
	Bangladesh	*P. ostreatus*		0.21 ± 0.00–0.30 ± 0.01	[31]
		*A. bisporus*		0.23 ± 0.01–0.30 ± 0.01	
		*V. volvacea*	0.24 ± 0.01		
		*G. lucidum*	0.21 ± 0.01		
	Mexico	*P. ostreatus*	63.0		[39]
		*A. bisporus (caps)*	4.06		
	Turkey	*P. sajor-caju*	8.50		[41]
Co	Bangladesh	*P. high-king*	0.011 ± 0.004	0.005–0.022	This study
		*P. ostreatus*	0.013 ± 0.005	0.005–0.024	
	Turkey	*P. sajor-caju*	12.5		[41]
	Australia	*A. bisporus*	0.126 ± 0.001		[40]
Cu	Bangladesh	*P. high-king*	14.2 ± 4.9	7.3–24.3	This study
		*P. ostreatus*	13.2 ± 3.7	6.7–18.8	
	Bangladesh	*P. ostreatus*		39.2 ± 0.88–102.1 ± 2.6	[31]
		*A. bisporus*		54.6 ± 0.86–163.4 ± 3.9	
		*V. volvacea*	101.8 ± 2.3		
		*G. lucidum*	72.5 ± 1.22		
	Turkey	*P. sajor-caju*	10.5		[41]
	Mexico	*P. ostreatus*	732		[39]
		*A. bisporus (caps)*	352		
	Australia	*A. bisporus*	53.4 ± 0.251		[40]
	Hungary	*A. bisporus*	58 ± 2–65 ± 1		[42]
Pb	Bangladesh	*P. high-king*	0.40 ± 0.39	0.08–1.37	This study
		*P. ostreatus*	0.22 ± 0.13	0.079–0.72	
	Bangladesh	*P. ostreatus*		0.14 ± 0.02–0.59 ± 0.03	[31]
		*A. bisporus*		0.15 ± 0.01–0.22 ± 0.02	
		*V. volvacea*	0.25 ± 0.02		
		*G. lucidum*	0.13 ± 0.01		
	Turkey	*P. sajor-caju*	27.5		[41]
	Mexico	*P. ostreatus*	0.91		[39]
		*A. bisporus (caps)*	0.41		
	Australia	*A bisporus*	3.9 ± 0.49		[40]
Mn	Bangladesh	*P. high-king*	11.2 ± 2.17	6.45–13.4	This study
		*P. ostreatus*	12.2 ± 2.4	5.1–15.8	
	Bangladesh	*P. ostreatus*		52.9 ± 1.04–104.5 ± 1.8	[31]
		*A. bisporus*		56.2 ± 1.34–91.1 ± 1.38	
		*V. volvacea*	78.5 ± 0.97		
		*G. lucidum*	64.0 ± 0.92		
	Turkey	*P. sajor-caju*	17.5		[41]
	Mexico	*P. ostreatus*	18.3		[39]
		*A. bisporus (caps)*	9.42		
	Australia	*A. bisporus*	10.6 ± 0.01		[40]
Hg	Bangladesh	*P. high-king*	0.126 ± 0.03	0.11–0.14	This study
		*P. ostreatus*	0.124 ± 0.03	0.094–0.15	
	Poland	*P. ostreatus*	0.10 ± 0.01 (cap), 0.02 ± 0.01 (stipe)		[18]
		*A. bisporus*	0.08 ± 0.01 (cap), 0.05 ± 0.01 (stipe)		
		*H. erinaceus*	0.07 ± 0.02 (cap), 0.03 ± 0.01 (stipe)		
Ni	Bangladesh	*P. high-king*	0.263 ± 0.18	0.12–0.95	This study
		*P. ostreatus*	0.3 ± 0.12	0.136–0.665	
	Turkey	*P. sajor-caju*	17.5		[41]
	Mexico	*P. ostreatus*	31.5		[39]
		*A. bisporus (caps)*	9.02		
Zn	Bangladesh	*P. high-king*	56.9 ± 19.5	26.3–85.8	This study
		*P. ostreatus*	50.2 ± 11.3	27.5–63.2	
	Bangladesh	*P. ostreatus*		30.1 ± 0.19–75.5 ± 0.54	[31]
		*A. bisporus*		36.3 ± 0.23–47.6 ± 0.46	
		*V. volvacea*	36.5 ± 0.43		
		*G. lucidum*	52.2 ± 0.47		
	Turkey	*P. sajor-caju*	110		[41]
	Ethiopia	*P. ostreatus*	59.2 ± 0.02		[43]
		*P. sajor-caju*	59.2 ± 0.03		
	Australia	*A. bisporus*	43.8 ± 0.20		[40]
	Hungary	*A. bisporus*	60 ± 0–62 ± 0		[42]

BDL: below detection limit (level 0.01 mg/kg 1 dw), LD: limit of detection, P: PleurotusL: Lentinula, A: Agaricus, T: Termitomyces, V: Volvariella, G: Ganoderma, H: *Hericium.*

**Table 3:** Accumulation factors of elements from substrate to mushrooms.

Location	Mushroom	As	Cd	Cr	Co	Cu	Pb	Mn	Hg	Ni	Zn
Savar	*P. high-king*	1.35	1.30	0.132	0.009	3.28	0.073	0.008	1.63	0.058	1.18
	*P. ostreatus*	1.54	3.12	0.249	0.038	2.03	0.090	0.016	0.82	0.047	1.01
Gazipur	*P. high-king*	0.79	2.11	0.293	0.032	1.61	0.271	0.012	0.79	0.042	0.76
	*P. ostreatus*	0.98	2.19	0.378	0.032	1.42	0.121	0.013	0.58	0.05	0.92
Mean		1.07	2.19	0.263	0.028	2.09	0.139	0.012	0.956	0.049	0.97
SD		0.51	1.19	0.189	0.020	1.13	0.169	0.006	0.754	0.020	0.43

**Table 4:** Correlation coefficient matrix of elements for mushroom samples.

	As	Cd	Cr	Co	Cu	Pb	Mn	Hg	Ni	Zn
As	1.000									
Cd	−0.508 **	1.000								
Cr	−0.016 ns	0.343 ns	1.000							
Co	−0.119 ns	0.132 ns	0.504 **	1.000						
Cu	0.778 **	−0.056 ns	0.170 ns	0.130 ns	1.000					
Pb	−0.312 ns	0.351 ns	0.679 **	0.522 **	−0.288 ns	1.000				
Mn	0.552 **	0.177 ns	0.312 ns	0.234 ns	0.645 **	−0.004 ns	1.000			
Hg	0.891 **	−0.470 *	−0.158 ns	−0.259 ns	0.666 **	−0.383 ns	0.352 ns	1.000		
Ni	−0.055 ns	0.522 **	0.215 ns	0.259 ns	0.117 ns	0.290 ns	0.154 ns	0.027 ns	1.000	
Zn	0.956 **	−0.322 ns	0.121 ns	−0.044 ns	0.806 **	−0.168 ns	0.699 **	0.826 **	0.075 ns	1.000

** and * denote *p* ≤ 0.01 and *p* ≤ 0.05, respectively; ns = non-significant.

**Table 5:** Health risks assessment for As and other elements from intake of mushroom for adults.

Element	Weekly Intake Rate (Fresh wt, g)	Median Concentration wt (mg/kg)	Weekly Intake (mg) of Elements	Weekly Intake of Element mg/kg/bw	Oral Reference Dose mg/kg/day	Oral Reference Dose mg/kg/week	HRI	PTWI µg/kg	PTWI mg/45 kg	Maximum Consumption per Week * (g)
As	0.09	0.047	0.0042	0.00009	0.0003	0.0021	0.0448	15	0.675	14.4
Cd		0.034	0.0030	0.00007	0.001	0.007	0.0097	7	0.315	9.3
Cr		0.027	0.0024	0.00005	0.003	0.021	0.0026			
Co		0.001	0.0001	0.00000						
Cu		1.250	0.1125	0.00250	0.04	0.28	0.0089			
Pb		0.021	0.0019	0.00004	0.004	0.028	0.0015	25	1.125	53.6
Mn		1.240	0.1116	0.00248	0.14	0.98	0.0025			
Hg		0.012	0.0011	0.00002				5	0.225	18.8
Ni		0.026	0.0023	0.00005	0.02	0.14	0.0004			
Zn		5.260	0.4734	0.01052	0.3	2.1	0.0050			

* Consumption rate of mushroom to reach provisional tolerable intake, g/week.
